# Pustular psoriasis of pregnancy in early first trimester: A case report

**DOI:** 10.1002/ccr3.4438

**Published:** 2021-07-21

**Authors:** Fatemeh Mohaghegh, Hamid Galehdari, Mina Rezaie

**Affiliations:** ^1^ Dermatology Department School of Medicine Isfahan University of Medical Sciences Isfahan Iran; ^2^ School of Medicine Isfahan University of Medical Sciences Isfahan Iran

**Keywords:** pustular psoriasis of pregnancy, first trimester, impetigo herpetiformis

## Abstract

Pustular psoriasis of pregnancy is a rare skin condition which mostly affects women in the third trimester and is sometimes followed by adverse outcomes for the mother and the fetus.

## Graphical abstract

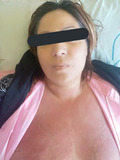

## 1 BACKGROUND

Pustular psoriasis of pregnancy (PPP) is a rare skin condition mostly in third trimester of pregnancy and is reportedly associated with adverse fetal outcomes. We report a case of generalized pruritic rash at 5 weeks gestation which led to a miscarriage 5 weeks later.

Skin changes during pregnancy can be classified as physiologic changes and dermatoses of pregnancy. There are several dermatoses that occur during pregnancy through postpartum period, including polymorphic eruption of pregnancy, pemphigoid gestationis and atopic eruption of pregnancy. Pruritus due to intrahepatic cholestasis of pregnancy leads to nonspecific skin lesions, including excoriation due to scratching. Pustular psoriasis of pregnancy (PPP), previously known as impetigo herpetiformis, represents generalized erythematous plaques with sheaths of pustules usually sparing the hands, feet, and face, and it may be related to the relative hypocalcemia of pregnancy.^1^ Skin lesions in PPP typically appear in the third trimester and resolve after delivery; however, lesions may be persistent through the postpartum period.^2^ Diagnosis of PPP is mostly clinical with the support of typical findings such as leukocytosis, elevated ESR, hypocalcemia and hypoalbuminemia. Typical histologic findings include parakeratosis, acanthosis, intraepidermal spongiform pustules containing neutrophils, and papillary dermal infiltration of lymphocytes and neutrophils.^3^, ^4^ There are reported cases of stillbirth, fetal anomalies, and neonatal deaths in PPP^5^, ^6^ which makes the diagnosis and management of PPP critical. Here, we report a patient with PPP at 5 weeks gestation who received adequate treatment which led to partial recovery of the skin lesions. After having a miscarriage at 10 weeks gestation and tapering the dose of corticosteroids, her skin lesions recurred.

## 2 CASE REPORT

**FIGURE 1:**
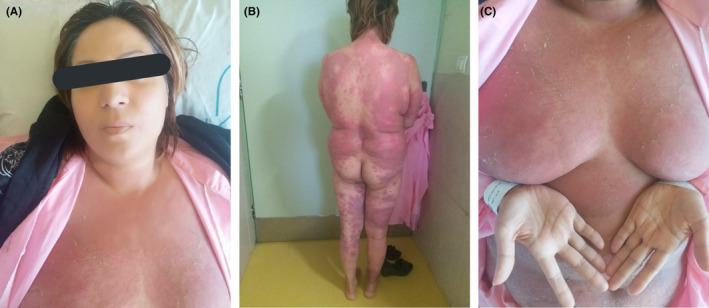
(A) Pustular lesions involving chest and abdomen and sparing the face. (B) Pustular lesions involving buttocks and back. (C) Spared palms

An otherwise healthy 35‐year‐old G1P0 woman (totally unaware of her pregnancy) presented to our dermatology department with a 2 weeks history of pruritic painful skin lesions. Her condition started with a single erythematous rash on the abdominal area. Following an assessment by a general practitioner, she received cloxacillin capsules, hydrocortisone injection, and mupirocin ointment which led to temporary recovery of the rash. After 3 days, more pruritic and painful skin lesions appeared on the trunk, upper and lower limbs. She had a history of a similar condition on her foot ankle about a year ago which did not spread and responded properly to topical treatment by steroid ointments. She was not currently taking any medications and denied having any systemic diseases. She had a history of irregular menstruation, so she was ignorant about her missing periods. On physical examination, erythematous plaques with pustules on the margins and surface with central scaling and variable size within 2 × 2‐cm to 3 × 3 mm involving the arms, chest, abdomen, back, groins, buttocks, and legs were noted. Older lesions had a central rim of desquamative scaling (Figure 1). Her face, sole of the feet, and palms were not involved, and oral mucosa was spared.

**FIGURE 2:**
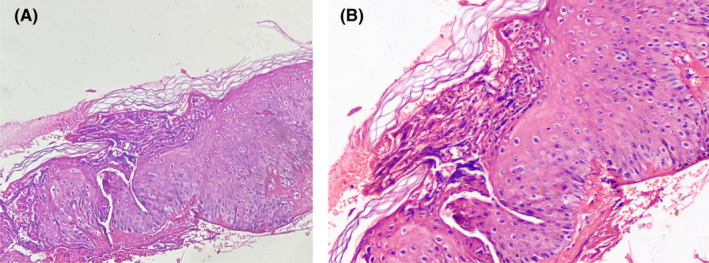
(A) Histopathology of a skin biopsy shows subcorneal pustule with mild acanthosis of epidermis (H&E ×100). (B) (H&E ×400)

Patient was admitted to dermatology department. Laboratory workup revealed leukocytosis alongside with other findings (Table 1). Since the initial diagnosis was generalized pustular psoriasis, we decided to initiate treatment with methotrexate. For this matter, we checked β‐hCG which was reported 3681 mIU/mL. After consulting the gynecology and obstetrician department, β‐hCG was checked again and a transvaginal sonography was operated reporting an intrauterine pregnancy sac without fetal pole and an estimated gestational age of 5 weeks + 4 days. Given the circumstances, our diagnosis was changed to pustular psoriasis of pregnancy which was supported by the pathologic findings as biopsy specimen revealed subcorneal pustules containing neutrophils, mild acanthosis of epidermis, and neutrophilic infiltration in papillary dermis (Figure 2).

**TABLE 1:** Laboratory findings

	Results	Reference range
WBC	13,000/mm^3^	4400–11,000/mm^3^
Neutrophil	83.2%	50%–70%
ESR (Erythrocyte sediment rate)	77 mm/h	0–20 mm/h
CRP (C‐reactive protein)	79 mg/L	1–6 mg/L
Albumin	2.9 mg/dL	3.9–4.9 mg/dL
Corrected calcium level	8.5 mg/dL	8.6–10 mg/dL

We began treatment with prednisolone with a dosage of 30 mg per day. During hospital admission, the patient complained of vaginal bleeding seeking gynecology care. A second transvaginal sonography was done reporting an intrauterine sac without fetal pole. It was recommended to repeat transvaginal sonography in 7–11 days. Having no history of diabetes or detected high blood glucose, our patient had high fasting blood sugars (FBS) and 3+ glucosuria in urinalysis repeatedly. She was diagnosed with diabetes and received insulin therapy under the observation of endocrinology department.

After partial recovery of skin rash, patient was discharged from hospital. She was observed by an OBGYN specialist. Following another episode of vaginal bleeding, it was confirmed by sonography that she had a miscarriage at the gestational age of 10 weeks. After the miscarriage, prednisolone dosage was tapered to 15 mg per day. Afterward, she developed new skin lesions, and due to the recurrence of the condition, we started cyclosporine for the patient. She is currently under treatment with cyclosporine, prednisolone, and insulin, and no new skin lesions have been reported afterward.

## 3 DISCUSSION

Pustular psoriasis of pregnancy or impetigo herpetiformis is a rare condition of unknown etiology which affects pregnant women mostly during the third trimester or in the postpartum period.^7^ In a 3‐year longitudinal study, it was accounted for 4.25% of all pregnancy dermatoses seen.^8^ It was first described by von Hebra et al, with a report of five pregnant women with pustular grouped lesions, with inflammatory nature and crust evolution, which all evolved into fetal deaths, in addition to four maternal deaths.^9^

The typical presentation of PPP is characterized by lesions that initially develop in skin folds with centrifugal spread.^6^ The lesions usually begin as erythematous plaques with a pustular ring with central erosion. The face, palms, and sole of the feet are typically spared with occasional involvement of oral and esophageal mucosae. Biopsy findings typically include spongiform pustules with neutrophil invasion into the epidermis. Typical laboratory findings include electrolyte derangements with elevated ESR and leukocytosis.^2^ Differential diagnoses for PPP include dermatitis herpetiformis, erythema multiforme, pustular subcorneal dermatosis, gestational pemphigoid, and acute generalized exanthematous pustulosis (AGEP).^10^, ^11^

The treatment of choice for PPP is systemic corticosteroids, with 30–60 mg of prednisolone per day. Cyclosporine may be used in refractory cases.^12^ Methotrexate and retinoids are contraindicated.^11^, ^13^ Although skin lesions in PPP are sterile, adjuvant therapy with antibiotics can be beneficial especially in cases of slight improvement after steroid therapy. Although lesions tend to disappear after delivery, there is a risk of recurrence in subsequent pregnancies, presenting earlier, with greater severity and worse maternal‐fetal prognosis. Currently, using systemic steroids and antibiotics has reduced maternal deaths considerably. However, the risk of stillbirth and perinatal mortality remains high, due to placental insufficiency, premature rupture of membranes, preterm labor, and intrauterine growth restriction.^10^

In our patient, the diagnosis of PPP was supported by the presence of erythematous plaques with pustules and central scaling alongside with pathologic findings of subcorneal pustules containing neutrophils and spongiosis. Although PPP normally occurs during the third trimester of pregnancy, rare cases have been reported as early as the first trimester of pregnancy.^14^ All sonographies before miscarriage reported a pregnancy sac without fetal pole, so there might be a slight possibility that the entire condition occurred due to blighted ovum. Although the patient received adequate treatments, she did not fully recover and skin lesions recurred after the miscarriage. Previous case reports also show that the skin lesions in PPP may be persistent through postpartum period.^2^

## 4 CONCLUSION

Pustular psoriasis of pregnancy (PPP) is a rare dermatosis with potential serious consequences for the mother and the child. Dermatologists and obstetricians must cooperate to improve the quality of life of the mother and contribute to a favorable outcome for the fetus.

## CONFLICT OF INTEREST

None declared.

## AUTHOR CONTRIBUTION

All the authors listed in the manuscript have participated actively and equally in presenting the case and providing the final version of the manuscript.

## ETHICAL APPROVAL

Written consent was taken from the patient for publishing the case including images. This case report was approved by the Bioethics committee of Isfahan University of Medical Sciences.

## Data Availability

No dataset was generated or analyzed during this case report.
